# Multimorbidity and its associated factors among adults aged 50 and over: A cross-sectional study in 17 European countries

**DOI:** 10.1371/journal.pone.0246623

**Published:** 2021-02-11

**Authors:** Dyego Leandro Bezerra de Souza, Albert Oliveras-Fabregas, Albert Espelt, Marina Bosque-Prous, Marianna de Camargo Cancela, Ester Teixidó-Compañó, Javier Jerez-Roig

**Affiliations:** 1 Department of Collective Health, Graduate Programme in Collective Health, Federal University of Rio Grande do Norte, Natal, Brazil; 2 Postgraduate Programme in Collective Health, Federal University of Rio Grande do Norte (UFRN), Natal-RN, Brazil; 3 Faculty of Health Sciences and Welfare, Research group on Methodology, Methods, Models and Outcomes of Health and Social Sciences (M_3_O), Centre for Health and Social Care Research (CESS), University of Vic-Central University of Catalonia (UVic-UCC), Barcelona, Spain; 4 Faculty of Psychology, Education and Sport Sciences Blanquerna, Physical Activity, Sport and Health Research Group, Universitat Ramon Llull, Barcelona, Spain; 5 Faculty of Health Sciences of Manresa, University of Vic–Central University of Catalonia, Manresa, Spain; 6 Departament de Psicobiologia i Metodologia en Ciències de la Salut, Universitat Autònoma de Barcelona (UAB), Bellaterra, Spain; 7 Centro de Investigación Biomédica en Red de Epidemiología y Salud Pública (CIBERESP), Madrid, Spain; 8 Faculty of Health Sciences, Universitat Oberta de Catalunya, Barcelona, Spain; 9 Brazilian National Cancer Institute, Rio de Janeiro, Brazil; Sciensano, BELGIUM

## Abstract

**Aims:**

To estimate the prevalence of multimorbidity among European community-dwelling adults, as well as to analyse the association with gender, age, education, self-rated health, loneliness, quality of life, size of social network, Body Mass Index (BMI) and disability.

**Methods:**

A cross-sectional study based on wave 6 (2015) of the Survey of Health, Ageing and Retirement in Europe (SHARE) was conducted, and community-dwelling participants aged 50+ (n = 63,844) from 17 European countries were selected. Multimorbidity was defined as presenting two or more health conditions. The independent variables were gender, age group, educational level, self-rated health, loneliness, size of network, quality of life, BMI and disability (1+ limitations of basic activities of daily living). Poisson regression models with robust variance were fit for bivariate and multivariate analysis.

**Results:**

The prevalence of multimorbidity was 28.2% (confidence interval–CI 95%: 27.5.8–29.0) among men and 34.5% (CI95%: 34.1–35.4) among women. The most common health conditions were cardiometabolic and osteoarticular diseases in both genders, and emotional disorders in younger women. A large variability in the prevalence of multimorbidity in European countries was verified, even between countries of the same region.

**Conclusions:**

Multimorbidity was associated with sociodemographic and physical characteristics, self-rated health, quality of life and loneliness.

## Introduction

Population ageing and changes in patterns of exposure to risk factors have increased the number of people living with chronic conditions [1]. The presence of two or more concomitant chronic diseases characterises multimorbidity, which is associated with negative consequences, mainly in relation to the limitation of functional capacity, well-being and social engagement [2].

Due to population ageing, the European continent has experienced a rise in costs given the increased demand for health services owing to the increase in patients with multimorbidity. The need to prioritise measures aimed at improving the follow-up of these patients becomes important for public health actions. Therefore, it is necessary to understand the distribution of multimorbidity in populations and their consequences over time [1].

Several studies describe the prevalence of multimorbidity in European countries. However, differences in prevalence need to be studied in detail by gender and age groups. According to a recent systematic review, in 58% of the prevalence studies the analysis was not based on age-group [3]. In addition, it is necessary to analyse which diseases occur more frequently among people with multimorbidity and what combinations of conditions are more common, in order to identify the different patterns of multimorbidity [4,5]. Identifying the patterns of multimorbidity is important for public policies, because this condition increases the consumption of drugs as well as the use and costs of health services, affecting the population’s quality of life [6].

In relation to the determinants of multimorbidity, there are still several aspects that need further studies, mainly regarding its risk factors, epidemiology, as well as the effectiveness of interventions [7]. The literature indicates an association with ageing, female gender, low socioeconomic status and more use of health services [5]. In addition, findings reveal that a higher Body Mass Index (BMI) and disability are associated with multimorbidity in different populations [8,9].

Another aspect that needs to be better studied is the association of multimorbidity with social environment. Poor quality of life has been associated with multimorbidity [10–12], while indicators of interaction and social participation, such as social network, social support and loneliness, were less studied. The evidence indicates an inverse association with social network and a direct (positive) association with loneliness [13].

Many ageing policies recommend the participation of people over 50 years old in leisure activities. However, there is less engagement of people with multimorbidity. Thus, it is fundamental that these policies to improve well-being and quality of life are based on strengthening social aspects, aimed at networks of friends, as well as on improving physical conditions and reducing psychological factors that may affect well-being [14]. Therefore, the relation between physical and social conditions and multimorbidity should be studied to support the expansion of social and health policies, since it will be possible to develop health promotion actions with better results [15,16].

Therefore, the present study sought to estimate the prevalence of multimorbidity among European community-dwelling adults aged 50 and over, as well as to analyse the association with gender, age, education, self-rated health, loneliness, quality of life, size of social network, Body Mass Index (BMI) and disability, as well as to analyse the differences in the profile of multimorbidity.

## Materials and methods

A cross-sectional population-based study was carried out using data from wave 6 release 7.0.0 of the Survey of Health, Ageing and Retirement in Europe (SHARE), the first multidisciplinary, cross-country, longitudinal research project conducted in Europe [17]. The SHARE project is subject to continuous ethics review and wave 6 was approved by the Ethics Council of the Max Planck Society and respects the Helsinki Declaration, in terms of anonymity of the participants and obtaining written consent [18]. Further details about the data collection, sampling procedures and other methodology aspects are available on the website www.share-project.org.

Data from individuals of the following 17 European countries were included: Austria, Belgium, Croatia, Czech Republic, Denmark, Estonia, France, Germany, Greece, Italy, Luxembourg, Poland, Portugal, Spain, Sweden, Slovenia and Switzerland. Although sampling differed slightly, all countries obtained probabilistic samples [18]. We used data from wave 6 (2015) of people aged 50 and over living in Europe. The total sample was of 65,160 individuals. For our study, we excluded people living temporarily or permanently in a nursing home, those with missing data in that variable, those with no data about multimorbidity and those with missing data to apply sampling weights (n = 1,316; 2%). The study sample was of 63,844 individuals. The average response rate was about 43% [19]. In this respect, the SHARE survey is no exception to the general decline in response rates in face-to-face surveys in Europe and worldwide [20].

The dependent variable “multimorbidity” was defined as the coexistence of two or more chronic conditions [5]. Thirteen non-communicable diseases or conditions were included: high blood pressure or hypertension (1), diabetes or high blood sugar (2), heart attack—including myocardial infarction, coronary thrombosis and any other heart problem including congestive heart failure (3), stroke (4), osteoarthritis (5), rheumatoid arthritis (6), cancer or malignant tumor—excluding minor skin cancers (7), chronic lung disease (8), hip fracture or femoral fracture (9), Parkinson’s disease (10), Alzheimer’s disease, dementia or senility (11), affective or emotional disorders—including anxiety, nervous or psychiatric problems (12) and chronic kidney disease (13). Information about non-communicable diseases was obtained through self-report. Conditions with little clinical repercussion and which are usually not included in multimorbidity studies were excluded [21]: high blood cholesterol, stomach or duodenal ulcer, cataracts and other fractures. Individuals with missing values in the dependent variable of the study were excluded from the regression analyses.

The independent variable “education” was determined using the International Standard Classification of Education (ISCED-97), which is a seven-point scale that standardises educational levels across Europe. For this study, the variable was re-categorised into three levels: low (ISCED-97 codes 0, 1 and 2); medium (ISCED-97 codes 3 and 4); and high (ISCED-97 codes 5 and 6) [22].

Other independent variables used were gender (male/female); age (categorised into age groups 50–59, 60–69, 70–79 and 80 plus); self-rated health (dichotomized into excellent/very good/good and fair/poor); Body Mass Index–BMI (categorized into underweight, normal weight, overweight and obese). BMI was estimated from self-reported weight and height.

Disability was defined as presenting a limitation in one or more basic activities of daily living (BADL). As asked in the questionnaire, any difficulty due to a physical, mental, emotional or memory problem that lasted at least three months was considered. The BADL included were dressing, walking across a room, bathing or showering, eating, getting in or out of bed and using the toilet (including getting up or down) [23].

Quality of life was measured with a modified and validated SHARE version of the *Control*, *Autonomy*, *Self-realisation and Pleasure* (CASP-12) scale, a shortened version of CASP-19. This scale includes 12 items related to four dimesions: control, autonomy, self-realisation and pleasure. Answers to every question were on a 4-point Likert scale (often, sometimes, rarely and never), with higher scores indicating a higher level of quality of life. Answers to each item were added up and global results divided in tertiles. The third tertile was considered low quality of life and second and first tertile medium-high quality of life [24]. Loneliness was assessed by a 3-item short version of the loneliness scale devoloped by Hughes *et al*. (2004) and was dichotomised into the following categories, in accordance with Steptoe *et al*. (2013): not lonely (3–5 points) and lonely (6–9 points) [25,26]. The size of social network was measured with a minimum 0 and maximum 7 people and classified by the median into the caregories small (0 and 1) and large (2–7) [27,28]. For variables with missing data, we created an extra category named “NR/DK” (No response/Do not know), which included all individuals with no information in that variable.

Prevalence of multimorbidity by country and gender were calculated. Calibrated individual weights, provided in the SHARE dataset, were applied to the analyses [18]. The patterns of multimorbidity were studied using descriptive analyses of more frequent disease combinations and the rank of diseases in respondents with multimorbidity, by gender and age groups. To compare differences between the categories of independent variables, bivariate analysis was carried out using Poisson regression models with robust variance to obtain prevalence ratios (PR) and their confidence intervals at 95% [29,30]. Multilevel Poisson regression models with robust variance were fit for multivariate analysis, obtaining PR and its confidence intervals (CI) at 95%. Two levels were included in the model (country and individual). Statistical analysis was conducted using STATA 16.

## Results

Of the whole sample, 21,458 individuals presented multimorbidity (Table 1), i.e. a prevalence of 28.2% (95%CI: 27.5–29.0) among men and 34.8% (95%CI: 34.1–35.4) among women. In men, multimorbidity was most frequent in the Czech Republic, followed by Germany, Poland and Estonia. The prevalence of multimorbidity in women was higher in Portugal, followed by Croatia, Poland and the Czech Republic. The countries with the lowest prevalence of multimorbidity in both genders were Switzerland, Sweden, Italy and Denmark (Table 2).

**Table 1:** Characteristics of the sample of community-dwelling people aged 50 and over.

Variable	n (63,844)	%
*Gender*		
Male	28276	44.29
Female	35568	55.71
*Age groups*		
50–59 years	16067	25.17
60–69 years	22912	35.89
70–79 years	16563	25.94
80 + years	8302	13.00
*Multimorbidity*		
No	42386	66.39
Yes	21458	33.61
*Number of chronic diseases*		
0	21505	33.68
1	20881	32.71
2	12275	19.23
3	5742	8.99
4	2272	3.56
5	786	1.23
6	267	0.42
7	80	0.13
8	27	0.04
9	7	0.01
10	2	0.00
*Country*		
Austria	3271	5.12
Germany	4271	6.69
Sweden	3789	5.93
Spain	5443	8.53
Italy	5117	8.01
France	3787	5.93
Denmark	3583	5.61
Greece	4767	7.47
Switzerland	2711	4.25
Belgium	5496	8.61
Czech Republic	4690	7.35
Poland	1779	2.79
Luxembourg	1512	2.37
Portugal	1624	2.54
Slovenia	4135	6.48
Estonia	5456	8.55
Croatia	2413	3.78

**Table 2:** Total number of people and prevalence of multimorbidity per country and gender in community-dwelling people aged 50 and over.

Country	Male		Female		Total	
	n	%(95%CI)	n	%(95%CI)	n	%(95%CI)
Austria	1386	27.0 (24.2–29.9)	1885	29.4 (27.0–31.8)	3271	28.3 (26.5–30.1)
Belgium	2491	27.5 (25.4–29.6)	3005	35.6 (33.4–37.7)	5496	31.7 (30.3–33.3)
Croatia	1085	29.1 (26.3–32.1)	1328	43.6 (40.7–46.6)	2413	37.2 (35.1–39.3)
Czech Republic	1904	38.4 (34.0–43.0)	2786	40.5 (37.3–43.7)	4690	39.5 (36.9–42.3)
Denmark	1665	26.9 (24.7–29.2)	1918	27.6 (25.5–29.8)	3583	27.3 (25.8–28.8)
Estonia	2158	30.0 (28.0–32.1)	3298	40.1 (38.4–41.9)	5456	36.1 (34.7–37.5)
France	1640	28.0 (25.7–30.4)	2147	33.4 (31.2–35.5)	3787	30.9 (29.3–32.5)
Germany	2038	35.2 (33.0–37.6)	2233	38.5 (36.2–40.8)	4271	37.0 (35.4–38.6)
Greece	2112	24.9 (23.0–26.8)	2655	36.7 (34.8–38.7)	4767	31.2 (29.9–32.6)
Italy	2348	24.1 (22.1–26.2)	2769	31.0 (29.0–33.1)	5117	27.8 (26.4–29.4)
Luxembourg	695	27.0 (23.5–30.8)	817	32.4 (28.6–36.3)	1512	29.7 (27.1–32.5)
Poland	784	33.6 (29.9–37.5)	995	42.6 (39.2–46.1)	1779	38.7 (36.1–41.3)
Portugal	735	28.8 (22.8–35.7)	889	47.3 (40.7–53.9)	1624	39.1 (34.5–43.9)
Slovenia	1790	27.5 (25.2–30.0)	2345	31.9 (29.8–34.1)	4135	29.9 (28.3–31.5)
Spain	2459	29.5 (25.9–33.3)	2984	36.9 (33.7–40.3)	5443	33.5 (31.1–36.0)
Sweden	1751	21.8 (19.6–24.2)	2038	24.8 (22.7–27.1)	3789	23.4 (21.8–25.0)
Switzerland	1235	20.3 (17.9–22.8)	1476	19.7 (17.6–21.9)	2711	20.0 (18.4–21.6)

**Fig 1:**
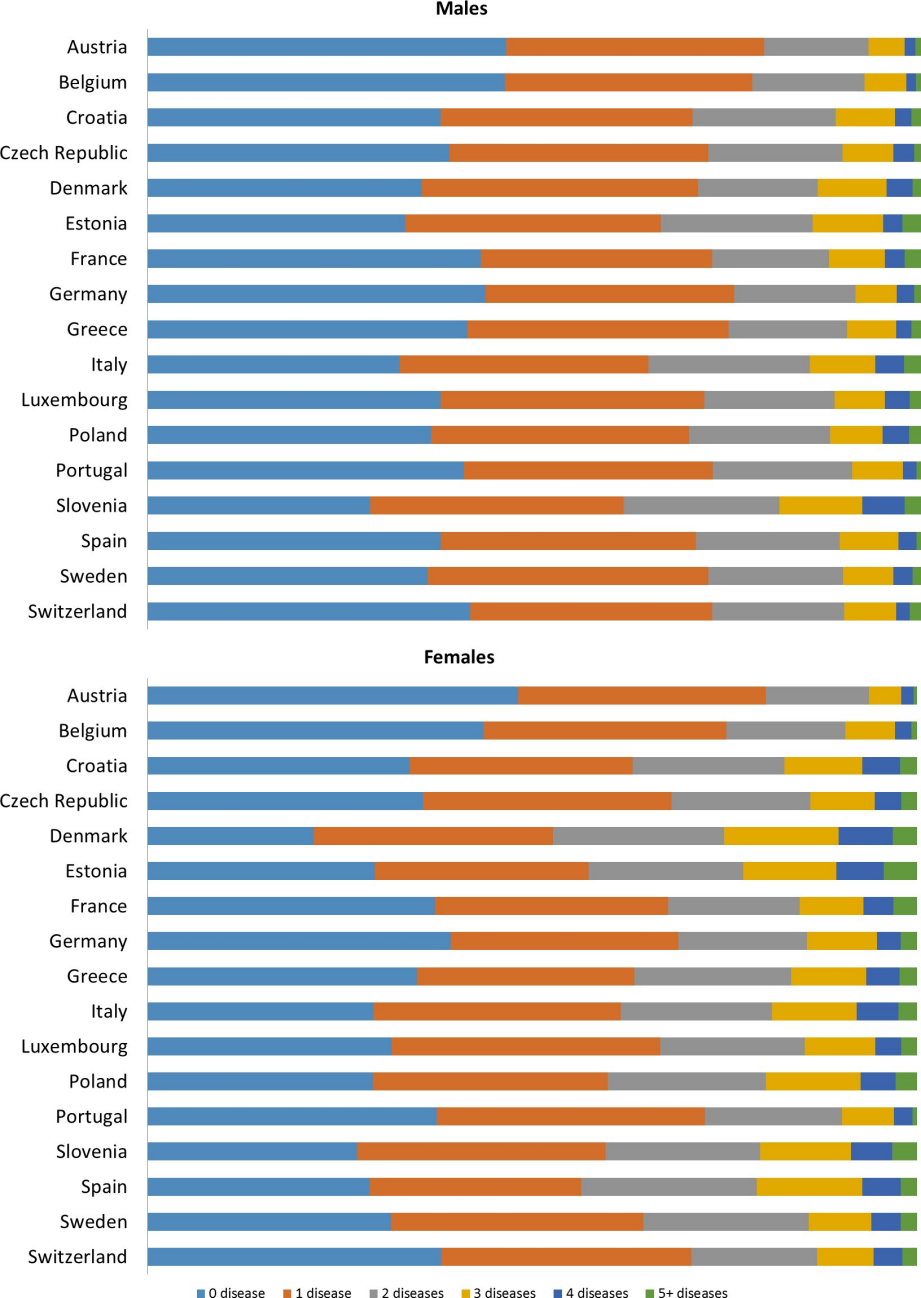
Prevalence of number of diseases per country and gender in community-dwelling people aged 50 and over. A) Males; and B) Females.

Fig 1 shows the prevalence of number of diseases by country in males and females. The Czech Republic, Portugal, Poland and Germany were countries that presented more prevalence of two, three or four diseases in males. In Portugal, Poland and Croatia more prevalence of two, three or four diseases in women was observed. The prevalence of five or more diseases was more common among women and with more variability than among men was observed.

Differences in the rank of diseases were identified when comparing men and women (Table 3). Hypertension, osteoarthritis, rheumatism, and emotional disorder were more frequent among women in the whole sample, whereas hypertension, heart attack and diabetes were more frequent in men.

**Table 3:** Rank of diseases in respondents by gender and age groups in community-dwelling people aged 50 and over.

Ranking	Age 50–59			
	Male		Female	
	Disease	% (95% CI)	Disease	% (95% CI)
**1**	Hypertension	27.4 (25.8–29.1)	Hypertension	24.4 (23.0–25.7)
**2**	Osteoarthritis	9.2 (8.1–10.3)	Osteoarthritis	14.6 (13.6–15.8)
**3**	Diabetes	8.6 (7.5–9.8)	Emotional disorder	9.8 (8.7–11.0)
**4**	Heart Attack	6.5 (5.6–7.5)	Rheumatism	7.0 (6.2–7.8)
**5**	Emotional disorder	4.8 (4.1–5.6)	Diabetes	5.5 (4.8–6.3)
**6**	Rheumatism	4.1 (3.4–4.8)	COPD	4.7 (4.1–5.3)
**7**	COPD	4.0 (3.4–4.7)	Cancer	3.4 (2.9–4.0)
**8**	Cancer	1.8 (1.5–2.3)	Heart Attack	3.1 (2.6–3.7)
**9**	Stroke	1.6 (1.3–2.0)	Kidney disease	1.5 (1.1–2.0)
**10**	Fracture	1.1 (0.8–1.5)	Stroke	1.4 (1.0–2.0)
**11**	Kidney disease	0.9 (0.6–1.3)	Fracture	0.6 (0.3–0.9)
**12**	Alzheimer	0.7 (0.3–1.5)	Alzheimer	0.4 (0.2–0.5)
**13**	Parkinson’s	0.4 (0.1–1.1)	Parkinson’s	0.1 (0.1–0.2)
	**Age 60–69**			
**1**	Hypertension	40.8 (39.7–41.9)	Hypertension	40.1 (39.1–41.1)
**2**	Diabetes	15.0 (14.2–15.8)	Osteoarthritis	23.2 (22.3–24.0)
**3**	Osteoarthritis	11.8 (11.1–12.4)	Diabetes	11.6 (11.0–12.3)
**4**	Heart Attack	11.4 (10.7–12.1)	Rheumatism	10.4 (9.8–11.1)
**5**	COPD	6.3 (5.8–6.8)	Emotional disorder	8.1 (7.5–8.7)
**6**	Rheumatism	5.4 (4.9–5.9)	Heart Attack	6.4 (5.9–6.9)
**7**	Cancer	4.2 (3.8–4.7)	COPD	5.6 (5.2–6.1)
**8**	Emotional disorder	4.0 (3.5–4.5)	Cancer	4.3 (3.9–4.7)
**9**	Stroke	3.5 (3.1–3.8)	Stroke	2.1 (1.9–2.4)
**10**	Kidney disease	1.5 (1.3–1.8)	Kidney disease	1.6 (1.4–1.9)
**11**	Fracture	1.0 (0.8–1.2)	Fracture	1.2 (1.0–1.4)
**12**	Alzheimer	0.7 (0.5–0.8)	Alzheimer	0.7 (0.5–0.8)
**13**	Parkinson’s	0.5 (0.4–0.6)	Parkinson’s	0.4 (0.3–0.5)
	**Age 70–79**			
**1**	Hypertension	49.0 (47.7–50.3)	Hypertension	54.4 (53.2–55.6)
**2**	Diabetes	19.5 (18.5–20.5)	Osteoarthritis	29.5 (28.4–30.6)
**3**	Heart Attack	18.0 (17.1–18.9)	Diabetes	16.8 (15.9–17.7)
**4**	Osteoarthritis	14.6 (13.7–15.5)	Rheumatism	15.0 (14.1–15.8)
**5**	COPD	8.5 (7.8–9.3)	Heart Attack	13.8 (13.0–14.7)
**6**	Rheumatism	7.1 (6.5–7.7)	Emotional disorder	8.4 (7.8–9.1)
**7**	Cancer	6.6 (6.0–7.2)	COPD	7.2 (6.6–7.9)
**8**	Stroke	6.0 (5.4–6.6)	Cancer	5.6 (5.1–6.2)
**9**	Emotional disorder	4.3 (3.8–4.8)	Stroke	3.8 (3.4–4.2)
**10**	Kidney disease	2.5 (2.1–2.9)	Fracture	2.6 (2.3–3.0)
**11**	Alzheimer	2.1 (1.8–2.4)	Kidney disease	2.5 (2.2–2.9)
**12**	Parkinson’s	1.8 (1.5–2.2)	Alzheimer	2.0 (1.7–2.4)
**13**	Fracture	1.6 (1.3–2.0)	Parkinson’s	1.3 (1.0–1.6)
	**Age 80+**			
**1**	Hypertension	48.5 (46.6–50.4)	Hypertension	55.7 (54.1–57.3)
**2**	Heart Attack	25.0 (23.4–26.7)	Osteoarthritis	33.5 (32.0–35.0)
**3**	Diabetes	18.3 (16.8–19.8)	Heart Attack	20.9 (19.6–22.2)
**4**	Osteoarthritis	17.9 (16.4–19.4)	Rheumatism	20.0 (18.7–21.3)
**5**	COPD	10.0 (8.8–11.2)	Diabetes	17.0 (15.8–18.3)
**6**	Rheumatism	9.1 (8.1–10.3)	Emotional disorder	10.1 (9.0–11.2)
**7**	Stroke	7.8 (6.9–8.9)	Alzheimer	8.4 (7.5–9.3)
**8**	Cancer	7.2 (6.2–8.3)	Stroke	7.0 (6.2–7.9)
**9**	Alzheimer	6.8 (5.9–7.8)	COPD	6.9 (6.1–7.7)
**10**	Emotional disorder	4.7 (3.8–5.7)	Fracture	5.7 (5.0–6.6)
**11**	Fracture	3.2 (2.5–4.2)	Cancer	4.1 (3.5–4.7)
**12**	Kidney disease	3.2 (2.7–3.9)	Kidney disease	3.7 (3.0–4.6)
**13**	Parkinson’s	2.5 (2.0–3.1)	Parkinson’s	1.9 (1.5–2.4)

COPD: Chronic obstructive pulmonary disease.

Regarding the number of chronic diseases in individuals that presented multimorbidity, 61.4% men and 54.6% women reported being diagnosed with two chronic diseases; 24.8% men and 27.5% women presented three; 9.4% men and 11.7% women presented four; and 4.4% men and 6.2% women reported having five or more chronic conditions (Table 4). It was observed that, among the individuals with multimorbidity, the most common combination was hypertension and diabetes in men and hypertension and osteoarthritis in women. Among those who reported three diseases, the most common combination was hypertension, diabetes and heart attack in men and hypertension, diabetes and osteoarthritis in women.

**Table 4:** Prevalence of number of diseases and disease combinations among community-dwelling people aged 50 and over with multimorbidity.

Number of diseases	% (95%CI)	Main combination of chronic diseases	
**Males**			
2	61.4 (60.0–62.8)	1^st^	Hypertension and diabetes
		2^nd^	Hypertension and heart attack
		3^rd^	Hypertension and osteoarthritis
		4^th^	Hypertension and COPD
		5^th^	Hypertension and rheumatism
3	24.8 (23.6–26.0)	1^st^	Hypertension, diabetes and heart attack
		2^nd^	Hypertension, diabetes and osteoarthritis
		3^rd^	Hypertension, osteoarthritis and heart attack
		4^th^	Hypertension, rheumatism and osteoarthritis
		5^th^	Hypertension, hearth attack and COPD
4	9.4 (8.5–10.4)	1^st^	Hypertension, heart attack, diabetes and osteoarthritis
		2^nd^	Hypertension, heart attack, stroke and diabetes
		3^rd^	Hypertension, heart attack, diabetes and COPD
		4^th^	Hypertension, diabetes, rheumatism and osteoarthritis
		5^th^	Hypertension, heart attack, rheumatism and osteoarthritis
5 or more	4.4 (3.9–5.0)	1^st^	Hypertension, diabetes, heart attack, cancer and osteoarthritis
		2^nd^	Hypertension, diabetes, heart attack, osteoarthritis and COPD
		3^rd^	Hypertension, heart attack, diabetes, rheumatism and osteoarthritis
		4^th^	Hypertension, heart attack, rheumatism, osteoarthritis and COPD
		5^th^	Hypertension, diabetes, heart attack, stroke and osteoarthritis
**Females**			
2	54.6 (53.5–55.7)	1^st^	Hypertension and osteoarthritis
		2^nd^	Hypertension and diabetes
		3^rd^	Hypertension and rheumatism
		4^th^	Hypertension and heart attack
		5^th^	Hypertension and emotional disorder
3	27.5 (26.6–28.5)	1^st^	Hypertension, diabetes and osteoarthritis
		2^nd^	Hypertension, rheumatism and osteoarthritis
		3^rd^	Hypertension, heart attack and osteoarthritis
		4^th^	Hypertension, osteoarthritis and emotional disorder
		5^th^	Hypertension, rheumatism and diabetes
4	11.7 (11.0–12.5)	1^st^	Hypertension, diabetes, rheumatism and osteoarthritis
		2^nd^	Hypertension, rheumatism, osteoarthritis and heart attack
		3^rd^	Hypertension, rheumatism, osteoarthritis and emotional disorder
		4^th^	Hypertension, diabetes, osteoarthritis and heart attack
		5^th^	Hypertension, osteoarthritis, heart attack and emotional disorder
5 or more	6.2 (5.6–6.8)	1^st^	Hypertension, diabetes, heart attack, rheumatism and osteoarthritis
		2^nd^	Hypertension, heart attack, rheumatism, arthritis and COPD
		3^rd^	Hypertension, diabetes, osteoarthritis, rheumatism and COPD
		4^th^	Hypertension, diabetes, rheumatism, emotional disorder and COPD
		5^th^	Hypertension, diabetes, osteoarthritis, rheumatism and emotional disorder

COPD: Chronic obstructive pulmonary disease.

According to our results, people with multimorbidity are more likely to be women, older, with lower education and self-reporting fair/poor health. Multimorbidity was also positively associated with higher BMI (overweight and obese), disability, high loneliness and low quality of life (Table 5).

**Table 5:** Bivariate and multivariate analysis of the associated factors with multimorbidity in community-dwelling people aged 50 and over (n = 51,988) of 17 European countries (2015).

	%(95%CI)	cPR(95%CI)	p-value	aPR(95%CI)	p-value
**Gender**					
Male	28.2 (27.5–29)	1		1	
Female	34.8 (34.1–35.4)	1.05 (1.03–1.06)	<0.001	1.02 (1.01–1.03)	<0.001
**Age (years)**					
50–59	17.3 (16.4–18.3)	1		1	
60–69	29.9 (29.2–30.5)	1.11 (1.09–1.12)	<0.001	1.08 (1.07–1.09)	<0.001
70–79	44.3 (43.4–45.1)	1.23 (1.20–1.26)	<0.001	1.16 (1.15–1.17)	<0.001
80+	53.5 (52.2–54.7)	1.31 (1.27–1.35)	<0.001	1.17 (1.15–1.19)	<0.001
**Educational level**					
High	22.6 (21.7–23.5)	1		1	
Medium	28.1 (27.3–28.8)	1.04 (1.03–1.05)	<0.001	1.00 (0.99–1.01)	0.589
Low	40.2 (39.4–41.1)	1.16 (1.14–1.18)	<0.001	1.01(1.01–1.02)	0.001
NR/DK	36.1 (32.2–40.2)	1.12 (1.07–1.17)	<0.001	1.00(0.99–1.01)	0.935
**Self-rated health**					
Excellent, very good or good	17.1 (16.6–17.5)	1		1	
Fair or poor	55.1 (54.2–55.9)	1.33 (1.31–1.35)	<0.001	1.22 (1.2–1.24)	<0.001
NR/DK	34.0 (9.9–70.9)	1.14 (0.92–1.41)	0.216	1.06 (0.84–1.34)	0.602
**Loneliness**					
Low	29.4 (28.9–29.9)	1		1	
High	52.4 (50.4–54.5)	1.18 (1.16–1.2)	<0.001	1.03 (1.02–1.04)	<0.001
NR/DK	53.8 (51–56.6)	1.18 (1.1–1.27)	<0.001	1.01 (1.00–1.03)	0.137
**Quality of Life**					
Medium-High	22.9 (22.3–23.4)	1		1	
Low	44.0 (43.1–44.9)	1.18 (1.16–1.20)	<0.001	1.05 (1.04–1.07)	<0.001
NR/DK	49.7 (47.4–52)	1.22 (1.15–1.29)	<0.001	1.05 (1.03–1.07)	<0.001
**Size of social network**					
Large	31.3 (30.3–32.3)	1			
Small	32.3 (31.7–32.9)	1.00 (0.99–1.02)	0.842		
Not asked	29.6 (28.2–31)	0.98 (0.92–1.04)	0.540		
**BMI**					
Normal	23.1 (22.4–23.8)	1		1	
Underweight	32.1 (28.2–36.3)	1.07 (1.05–1.11)	<0.001	0.99 (0.97–1.01)	0.285
Overweight	30.5 (29.8–31.3)	1.06 (1.05–1.07)	<0.001	1.05 (1.04–1.06)	<0.001
Obese	46.6 (45.4–47.8)	1.18 (1.17–1.2)	<0.001	1.14 (1.12–1.15)	<0.001
NR/DK	44.4 (40.9–47.9)	1.16 (1.13–1.2)	<0.001	1.03 (1.00–1.05)	0.023
**Disability**					
No	27.4 (26.9–27.9)	1		1	
Yes	66.1 (64.7–67.5)	1.30 (1.28–1.32)	<0.001	1.100 (1.08–1.11)	<0.001
NR/DK	26.9 (7.9–61.4)	1.00 (0.81–1.25)	0.982	0.89 (0.76–1.04)	0.149

95%CI: 95% Confidence Interval; PR: Prevalence Ratio; NR/DK: No response/Do not know; BMI: Body mass index.

## Discussion

This research presented the prevalence of multimorbidity in European community-dwelling adults aged 50 and over that reported two or more non-communicable conditions. As far as we know, this study has included more European countries than any previous study, and we have also conducted specific analyses by gender and age group. We observed that the prevalence of multimorbidity increases with age and was highest in women. The estimated prevalence of multimorbidity (approximately 33%) was similar to that found in wave 5 of the SHARE study [21]. However, we identified important differences between countries with the highest variation (almost 20%), corresponding to the one between Switzerland (20%) and the Czech Republic (39%). Large differences are found even in countries of the same European region, which indicates that there is no homogeneity that justifies a pooled analysis stratified by region. Garin *et al*. [4] analysed the prevalence in Finland, Poland, Spain, Russia, China, Ghana and South Africa and the higher prevalence was observed in high income countries. According to the authors, the higher prevalence has been associated with increased level of development.

When comparing data with other studies, it is necessary to make some methodological considerations. Firstly, the definition of multimorbidity is heterogeneous and there is much variety concerning which chronic conditions to include, which makes comparability between studies difficult [3,31]. The presence of two or more chronic conditions is the most common definition [32], but the number of included non-communicable diseases varies between 4 and 102 in the literature [16]. Another important aspect is related to the method used to collect the information; although some studies use medical records to identify diagnosed conditions, most authors consider information reported by the individual [5,15,33,34]. It is worth noting that self-reported data has the potential bias of underestimation of prevalence, but it is the most feasible method for population-based epidemiological studies [16].

As found in our results, several studies show that multimorbidity is more prevalent in women and increases with age [35,36]. However, many studies fail to address the differences in multimorbidity patterns considering these two aspects. It is necessary to analyse disease patterns stratified between men and women and how these patterns evolve with ageing. Therefore, variations exist when analysing these patterns by combining gender and age groups [3].

Multimorbidity patterns are increasingly studied through statistical techniques of interdependence, such as cluster or factorial analysis, or by analysing the frequency of combinations of the major diseases [37]. The most common patterns found in the literature are cardiovascular diseases combined with osteoarticular and metabolic diseases [35,38]. Our results show that younger women are more affected by mental and osteoarticular disorders than younger men, and stroke and diabetes are more prevalent conditions in men and increase with age. However, this interpretation needs to be made with caution since this is a cross-sectional study and a cohort effect may exist.

Interestingly, some modifiable social factors can positively influence health, such as social and affective networks and the maintenance of functional abilities, which help the patient to become more independent [39]. In line with these findings, we found that people with high loneliness and low quality of life have a higher prevalence of multimorbidity. Olaya *et al*. [13] studied social support and loneliness as prognostic factors in older adults with and without multimorbidity. Survival was reduced in people with low social support, but loneliness did not modify the effect of multimorbidity. It is important to emphasise that, due to the fact that chronic health issues are more frequent at more advanced ages, there is a need for strategies that promote the social participation of this population group [14].

Quality of life and loneliness are indicators that may reflect the socio-cultural environment of the individual. In this sense, Stumberg *et al*. [40] understand multimorbidity as a *complex adaptive systems response* to biobehavioural and socio-environmental networks. For them, multimorbidity is the manifestation of interconnected physiological network processes within an individual in a socio-cultural environment. Walker and Peterson [41] use the concept of *habitas* in a sociological perspective to see multimorbidity as being social, economic and a broader part of experiencing social systems. Therefore, the experience of illness of people living with multiple coexisting conditions develops with the *greater social system of health and illness*.

We also studied the physical characteristics and their relationship with multimorbidity and found an association with disability and overweight/obesity. These results corroborate the findings of other studies and highlight the importance of promotion and prevention actions to improve self-management during the aging process [2,8,9,42]. It is necessary to evaluate the influence of these strategies (including health policies) through longitudinal studies that follow the evolution of the incidence of multimorbidity, especially among those who are more vulnerable and socially isolated [14]. Therefore, individuals with multimorbidity may develop physical impairment and social isolation; this needs to be studied further, as it limits public health actions and highlights the importance of strategies to enhance physical activity and social engagement.

Another aspect to consider when analysing the factors associated with multimorbidity is the socioeconomic level. In line with our findings, other studies have verified a positive association with lower educational categories [43,44]. Studies that analysed economic variables found that multimorbidity is associated with lower income, with a lower prevalence in the high tertile income comparing different regions of Europe [21,36]. As a result, it is necessary to conduct an in-depth analysis of how other contextual variables can influence this relationship, e.g. type of work carried out throughout life, influence of social policies and quality of access to health services [7]. The differences in prevalence that we found between countries of the same European region, such as Portugal and Spain, probably reflect the differences in social policies, quality of socio-health services and the historical and cultural context of these countries. Another study carried out with SHARE data analysed health inequalities comparing the countries grouped in three political traditions. The differences in poor self-reported health between people with high and low education were largest in Late, Social and Christian democracies, respectively [45].

In this context, understanding the influence of social determinants of health becomes essential for the study of multimorbidity, in order to influence populations differently according to social class and life context. The association between multimorbidity and poor or fair self-rated health is another aspect that should be considered when planning interventions in patients with multimorbidity. This association was also found in other studies [31,36]. Self-knowledge of the condition of multimorbidity is probably a determining factor in assessing health as fair or poor.

The detailed study of multimorbidity reveals that it is vital to evaluate the efficacy of individual and collective interventions considering this multiplicity of standards. Intervention studies usually exclude individuals with multiple chronic conditions and at more advanced ages. Besides, health services need to act and treat these people and end up performing interventions based on studies that do not have external validity for this population group, which increases the risk of adverse events. Multimorbidity requires a shift from the traditional model of health care (with a focus on the disease) to an individualised care. Some authors even warn that there must be a change in the form of payment of medical procedures, which should consider the complexity of each case [7,46,47].

Regarding the limitations of our study, this is a cross-sectional study and therefore does not allow for an analysis of the direction of causal relations. Another aspect to consider is the sampling of the oldest age group: some studies with SHARE data did not use the data of individuals aged 80 and over due to lack of comparability across countries [23]. Similar to other studies, we decided to include this age group, and we did so for two reasons. First, due to their relevance to the issue; and second, because of the observation that, in wave 6, only Croatia obtained a proportion of this age group that is much greater than that found in the other countries. However, these difficulties are compensated by the harmonisation and standardisation of the questionnaires and data collection procedures, guaranteed by the meticulous process of cultural adaptation, as well as by the high professionalism of highly trained researchers and interviewers.

With regards to the strengths of our study, we highlight that this is the European prevalence study that includes more countries with detailed analysis of multimorbidity, specifying disease combinations and ranking diseases according to their frequency. In addition, one advantage of a longitudinal study such as SHARE is the quality and quantity of data of different waves and countries due to the standardisation of data collection, which strengthens the data when used in cross-sectional studies such as ours. Moreover, difficulties in conducting a large study with heterogeneous populations and countries should be recognised. The diseases/conditions we include to define multimorbidity may also be a limitation. However, we reaffirm that there is no standardisation under which conditions should be included and chose to include the same as other studies using SHARE data [16,21].

In conclusion, the estimates showed that there is large variability in the prevalence of multimorbidity in European countries, even between countries in the same region. The prevalence of multimorbidity, approximately 33%, was related to sociodemographic characteristics of the population, and increases and changes with ageing. Multimorbidity was most prevalent in the Czech Republic, Poland and Portugal. The countries with the lowest multimorbidity prevalence were Switzerland, Sweden and Denmark. The most common conditions were cardiometabolic and osteoarticular diseases in both genders, and emotional disorders in younger women. Multimorbidity was associated with sociodemographic and physical characteristics, poor self-rated health, low quality of life and loneliness. In this context, the focus on a single disease needs to be modified in order to provide better interventions, which should be continued and personalised considering the different patterns of multimorbidity and the historical, social and economic context of individuals. This may require changes in medical education, research, organisation and funding of health services.
